# Just a Flu? Self-perceived infection mediates the link between conspiracy beliefs and Covid-19 health beliefs and behaviors

**DOI:** 10.1177/13591053211051816

**Published:** 2021-10-20

**Authors:** Jan-Willem van Prooijen, Tom W Etienne, Yordan Kutiyski, André PM Krouwel

**Affiliations:** 1 Vrije Universiteit Amsterdam, The Netherlands; 2 The Netherlands Institute for the Study of Crime and Law Enforcement, The Netherlands; 3 Maastricht University, The Netherlands; 4 Kieskompas – Election Compass, The Netherlands

**Keywords:** conspiracy theories, Covid-19, health beliefs and behaviors, infection, institutional trust

## Abstract

The Covid-19 pandemic has inspired many conspiracy theories, which are associated with detrimental health beliefs and behaviors (e.g. reduced physical distancing; decreased vaccination intentions). We propose a previously unrecognized mediator of these relationships: A self-perceived likelihood to already have experienced a Covid-19 infection. Results from a large sample (*N* = 9033) revealed that self-perceived infections mediated the link between conspiracy beliefs and health beliefs and behaviors. These findings emerged independently of institutional distrust, and actual infections as indicated by a positive medical test. These findings suggest that conspiracy beliefs shape people’s interpretation of the physical signals of their own body.

The Covid-19 pandemic has elicited many conspiracy theories, such as that the corona virus was created by humans in a lab to destroy economies, that the corona virus is a hoax, or that the pandemic has been exaggerated deliberately by national governments to suppress the people. Conspiracy theories are defined as explanatory beliefs making assumptions that a group of actors collude in secret to pursue nefarious goals (Bale, 2007; van Prooijen, 2018, 2020). While many different conspiracy theories exist, beliefs in these different theories are typically positively correlated (Goertzel, 1994; Swami et al., 2011; Wood et al., 2012), suggesting a common underlying psychology that enables predictions of when conspiracy beliefs are more likely. For instance, conspiracy theories surge particularly against the background of societal crisis situations including wars, terrorist attacks, natural disasters, economic crises, and so on (van Prooijen and Douglas, 2017). Indeed, conspiracy theories are stimulated by aversive feelings that are naturally associated with crisis situations, such as anxiety, uncontrollability, and uncertainty (for overviews, see Butter and Knight, 2020; Douglas et al., 2019; Uscinski and Parent, 2014; van Prooijen and van Vugt, 2018). Moreover, big and impactful societal events elicit “consequence-cause matching,” that is, a tendency to assume there is a big cause—such as a major conspiracy—to explain such events (McCauley and Jacques, 1979; van Prooijen and van Dijk, 2014). As such, the Covid-19 pandemic has provided fertile soil for conspiracy theories to flourish.

Even when irrational, conspiracy theories can exert a genuine influence on a wide range of perceptions and behaviors (van Prooijen and Douglas, 2018). Consistently, accumulating research has revealed associations between conspiracy beliefs and health beliefs and behaviors that are relevant during the Covid-19 pandemic. For instance, conspiracy beliefs have been associated with decreased physical distancing, decreased support for restrictive measures to contain the virus, increased support for interventions not supported by science, decreased intentions to get vaccinated, and so on (Freeman et al., 2020; Hornsey et al., 2021; Imhoff and Lamberty, 2020; Marinthe et al., 2020). At least some of these findings represent a causal effect of conspiracy beliefs promoting detrimental health beliefs and behaviors (Pummerer et al., 2021). These findings are consistent with research carried out before the pandemic, underscoring that conspiracy theories can be consequential for health, as, for instance, reflected in decreased vaccination intentions (Jolley and Douglas, 2014). Moreover, studies in South Africa reveal that AIDS conspiracy beliefs (e.g. beliefs that the HIV virus was made by humans) predict reduced condom use among both men and women (Grebe and Nattrass, 2012).

An important question, however, is *why* conspiracy beliefs are associated with detrimental health beliefs and behaviors in the context of Covid-19. One common explanation is a link with institutional distrust, that is, negative expectations of institutions and therefore a preference to avoid being in a vulnerable position toward them. The basic idea is that ascribing malevolent plots to powerful institutions (including experts and elites) erodes the trust that people have in such institutions. As a consequence, people are less inclined to endorse the recommendations or policy solutions of these institutions; instead, people become susceptible to alternative—and potentially unsubstantiated—ideas about, for instance, the dangers of the coronavirus, and how to avoid infection. Empirical research indeed underscores a key role for institutional distrust in understanding the societal implications of Covid-19 conspiracy theories (Freeman et al., 2020; Karić and Međedović, 2021; Pummerer et al., 2021; Šrol et al., 2021).

The present contribution proposes an additional, previously unrecognized mediator between conspiracy beliefs and health perceptions and behaviors in the context of the Covid-19 pandemic: The belief that one already has been infected with the virus. Specifically, conspiracy thinking is associated with a more generally suspicious outlook on the world (e.g. Darwin et al., 2011; Imhoff and Lamberty, 2018). It therefore stands to reason that conspiracy beliefs are also associated with increased suspicion toward the physical signals of one’s own body, and to interpret a relatively mild discomfort as evidence of a Covid-19 infection. Some Covid-19 conspiracy theories have been consistently associated with feeling less threatened by the virus, and equating Covid-19 with seasonal flu (Imhoff and Lamberty, 2020). Such a belief system easily supports the conclusion that one has already experienced a Covid-19 infection, for instance after experiencing mild physical symptoms that more likely are caused by a common cold or a mild flu. Such self-perceived (and often unwarranted) signs of infection may reduce Covid-19 containment behavior in a myriad of ways: For instance, it may reinforce beliefs such as that Covid-19 produces only mild symptoms, or that treatments that one routinely uses against a common cold are effective against Covid-19; moreover, it may install a belief that one already has antibodies against the virus.

The current study investigated these issues through a large sample (weighted to provide nationally representative estimates of the Dutch adult population), collected at the beginning of the pandemic in the Netherlands (April 2020). The study included specific measures of Covid-19 conspiracy beliefs, but also a more general measure of conspiracy mentality—that is, a dispositional tendency to assume conspiracies are responsible for major events in the world (Bruder et al., 2013). Moreover, the study assessed a number of health beliefs relevant for the Covid-19 pandemic, including participants’ beliefs of how dangerous the virus is, their support for interventions supported by scientists (e.g. reducing public gatherings), and their support for interventions not supported by science (e.g. relying on herbal medicine to combat Covid-19). In addition, the study assessed health behaviors, including physical distancing, hygiene behavior, and vaccination intentions. We assessed institutional trust and self-perceived infections as mediators, to test whether self-perceived infections constitute a mediator of the link between conspiracy beliefs and health-relevant beliefs and behaviors independent of institutional trust. Finally, the study also assessed if people actually have been infected with the corona virus according to a positive medical test.

## Method

### Sample

The study was conducted on a large research panel by Election compass (“Kieskompas”), a Dutch political research organization that fully adheres to GDPR (i.e. EU privacy) regulations, is closely monitored by the Dutch privacy authority, and acts in line with the ethical norms of VU Amsterdam. The study also has formal ethical approval, and was carried out consistent with the provisions of the declaration of Helsinki. The study was part of a larger project that took place at the start of the pandemic in the Netherlands (April 2020; Krouwel et al., 2020). Participants gave their informed consent online. Prior to any analysis, the data were weighted to provide nationally representative population estimates through poststratification iterative proportional fitting, with benchmarks of age, sex, education, geographical region, ethnicity, and vote recall, relying on the Dutch golden standard (CBS), as well as the official 2017 parliamentary election results. The sample included 9033 participants (raw unweighted demographics, 6084 men, 2949 women; M_age_ = 55.36, SD = 15.94; after weighting, 4474 men, 4559 women; M_age_ = 49.10, SD = 17.43). This sample yields more than 99% power to detect even extremely small effect sizes (*f*^2^ = 0.002).

### Measures

A full overview of the items that formed the basis of this contribution is provided in the Supplemental Materials.

#### Conspiracy beliefs

To measure Covid-19 conspiracy beliefs, participants were asked to rate how credible they found nine relevant conspiracy theories (1 = *not very credible*, 5 = *very credible*), such as “The virus has been released by the US government to destabilize China,” and “The virus was developed to control population growth” (α = 0.89).^1^ Furthermore, we assessed the 5-item Conspiracy Mentality Questionnaire (Bruder et al., 2013; 1 = *certainly not 0%*, 11 = *certainly 100%*), including items such as “I think that many very important things happen in the world, that the public is never informed about” (α = 0.89).

#### Trust in institutions

Participants rated how much trust they had in 16 institutions (1 = *Not at all*, 4 = *A lot*). Most institutions were domestic and included the army, the educational system, the media, the police, and so on. The questionnaire also included a few international institutions (NATO; the European Union). Together, participants’ responses formed a reliable scale of institutional trust (α = 0.90).

#### Self-perceived and actual infections

To measure self-perceived infections, participants rated how big they considered the chance that they already were (or already had been) infected with the coronavirus (1 = *very small*; 5 = *very big*). We also measured actual infections by asking dichotomously if participants had received a positive medical test for Covid-19 (*no* vs *yes*).

#### Health beliefs and behaviors

As *health beliefs*, we measured perceived danger, participants’ support for interventions not endorsed by health scientists, and participants’ support for interventions endorsed by health scientists. Perceived danger was assessed with three items, including “It is dangerous to get infected with the coronavirus” (1 = *certainly not*, 5 = *certainly*; α = 0.63). Participants also rated the perceived effectiveness (1 = *not at all effective*, 5 = *very effective*) of eight interventions not recommended by health scientists, such as “ignore the virus and continue life as usual,” and “rely on herbal medicine or other alternative treatments” (“unscientific intervention support”; α = 0.70); moreover, participants rated the perceived effectiveness of eight interventions recommended by health scientists, including “Stay indoors as much as possible” and “Scale up the capacity of Intensive Care Units in hospitals” (“scientific intervention support”; α = 0.77).

As *health* behaviors, we measured physical distancing, personal hygiene, and vaccination intentions. Specifically, five items asked for participants’ physical distancing behavior, including “During the days of the corona pandemic, I stay at home as much as possible” (responses on a slider from 0 = *strongly disagree*, to 10 = *strongly agree*; α = 0.67). Another five items assessed participants’ personal hygiene behavior on the same slider, including “During the days of the corona pandemic, I wash my hands longer than usual” (α = 0.78). Finally, participants responded to the question “Would you get vaccinated with a future vaccine against Covid-19?” (1 = *certainly not*, 5 = *certainly*).

### Statistical analysis

**Figure 1.**
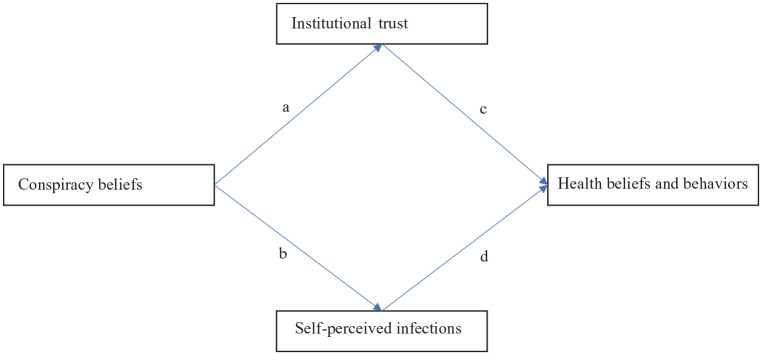
The relationship between conspiracy beliefs and Covid-19 health beliefs and behaviors through institutional trust and self-perceived infections: Parallel mediation model. Statistics for the a, b, c, and d-paths are in Table 2.

We test our line of reasoning through parallel mediation analyses using bootstrapping (Process model 4, 1000 samples, bias-corrected; Hayes, 2013). The resulting bias-corrected bootstrap intervals have been found to be highly reliable tests of indirect effects in mediation analyses (Hayes and Scharkow, 2013). The mediation model (displayed in Figure 1) included Covid-19 conspiracy beliefs and conspiracy mentality as independent variables, trust in institutions and self-perceived infections as parallel mediators, and health beliefs and behaviors as dependent variables. All analyses empirically controlled for gender, age, education, and political orientation (results were similar without these control variables).

### Data sharing

An anonymized version of the dataset, a code to reproduce the results, and the Supplemental Materials, are publicly available on OSF.^2^ The study was not preregistered.

## Results

Means, standard deviations, and intercorrelations are displayed in Table 1. Covid-19 conspiracy beliefs and conspiracy mentality were both associated with decreased institutional trust and an increased belief to already have been infected. Moreover, Covid-19 conspiracy beliefs and conspiracy mentality were associated with all the health-related beliefs and behaviors in the expected ways (although the correlation between Covid-19 conspiracy beliefs and hygiene behaviors was small). Table 2 displays the regression coefficients of the various paths in the mediation models.

**Table 1.** Means, standard deviations, and intercorrelations of the measured variables.

	M	SD	1	2	3	4	5	6	7	8	9	10
1. Covid-19 conspiracy beliefs	1.84	0.69	–									
2. Conspiracy mentality	5.25	2.15	0.53*	–								
3. Institutional trust	2.58	0.47	−0.33*	−0.41*	–							
4. Self-perceived infections	2.37	1.12	0.19*	0.12*	−0.12*	–						
5. Perceived danger	3.66	0.84	−0.24*	−0.10*	0.20*	−0.20*	–					
6. Unscientific intervention support	1.77	0.52	0.43*	0.29*	−0.23*	0.05*	0.13*	–				
7. Scientific intervention support	4.00	0.57	−0.31*	−0.18*	0.29*	−0.19*	0.51*	−0.18*	–			
8. Physical distancing behaviors	7.72	1.67	−0.13*	−0.08*	0.07*	−0.16*	0.40*	−0.05*	0.36*	–		
9. Hygiene behaviors	6.98	1.98	−0.03^†^	−0.09*	0.10*	−0.03^†^	0.28*	−0.01	0.38*	0.43*	–	
10. Vaccination intentions	4.08	1.15	−0.40*	−0.26*	0.30*	−0.19*	0.40*	−0.35*	0.44*	0.22*	0.20*	–

Higher scores indicate higher ratings on the variable in question. Institutional trust was measured on a 4-point scale (1–4); Conspiracy mentality (1–11), physical distancing, and hygiene behaviors (0–10) were measured on 11-point scales; all remaining variables were measured on 5-point scales (1–5).

* *p* < 0.001. ^†^*p* < 0.05.

**Table 2.** Results of parallel mediation analyses.

Covid-19 conspiracy beliefs	a-Path		b-Path	
	*B* (SE)	CI 95%	*B* (SE)	CI 95%
All dependent variables^a^	−0.257 (0.009)*	[−0.275; −0.239]	0.255 (.026)*	[0.206; 0.306]
	c-Path		d-Path	
	*B* (SE)	CI 95%	B (SE)	CI 95%
Perceived danger	0.327 (0.024)*	[0.280; 0.374]	−0.049 (0.009)*	[−0.066; −0.032]
Unscientific intervention support	−0.105 (0.013)*	[−0.131; −0.078]	0.026 (0.005)*	[0.017; 0.036]
Scientific intervention support	0.257 (0.015)*	[0.228; 0.287]	−0.022 (0.005)*	[−0.033; −0.012]
Physical distancing behaviors	0.218 (0.049)*	[0.121; 0.315]	−0.042 (0.018)^†^	[−0.077; −0.007]
Hygiene behaviors	0.403 (0.060)*	[0.285; 0.521]	0.050 (0.022)^†^	[0.007; 0.093]
Vaccination intentions	0.452 (0.030)*	[0.394; 0.511]	−0.046 (0.011)*	[−0.067; −0.025]
Conspiracy mentality	a-Path		b-Path	
	*B* (SE)	CI 95%	*B* (SE)	CI 95%
All dependent variables^a^	−0.076 (0.002)*	[−0.080; −0.072]	0.054 (0.006)*	[0.041; 0.066]
	c-Path		d-Path	
	*B* (SE)	CI 95%	*B* (SE)	CI 95%
Perceived danger	0.366 (0.025)*	[0.317; 0.414]	−0.054 (0.009)*	[−0.071; −0.037]
Unscientific intervention support	−0.138 (0.014)*	[−0.166; −0.110]	0.033 (0.005)*	[0.023; 0.043]
Scientific intervention support	0.292 (0.016)*	[0.262; 0.323]	−0.027 (0.006)*	[−0.038; −0.016]
Physical distancing behaviors	0.269 (0.051)*	[0.170; 0.369]	−0.050 (0.018)^†^	[−0.085; −0.015]
Hygiene behaviors	0.363 (0.062)*	[0.242; 0.485]	0.052 (0.022)^†^	[0.010; 0.095]
Vaccination intentions	0.514 (0.031)*	[0.453; 0.576]	−0.058 (0.011)*	[−0.079; −0.036]

The a-path represents the link between conspiracy beliefs and institutional trust, the b-path between conspiracy beliefs and self-perceived infections, the c-path between institutional trust and the dependent variables, and the d-path between self-perceived infections and the dependent variables.

a Exact statistics vary slightly at the third decimal across dependent variables due to missing values.

* *p* < 0.001. ^†^*p* < 0.05.

### Covid-19 conspiracy beliefs

The indirect effect through trust was significant for perceived danger, *B* = −0.084, SE = 0.008, CI_95%_[−0.010; −0.069]; for unscientific intervention support, *B* = 0.027, SE = 0.004, CI_95%_[0.019; 0.035]; for scientific intervention support, *B* = −0.066, SE = 0.006, CI_95%_[−0.078; −0.055]; for physical distancing behaviors, *B* = −0.056, SE = 0.014, CI_95%_[−0.083; −0.027]; for hygiene behaviors, *B* = −0.104, SE = 0.018, CI_95%_[−0.138; −0.067]; and for vaccination intentions, *B* = −0.116, SE = 0.010, CI_95%_[−0.135; −0.098]. The indirect effect through self-perceived infections was also significant for perceived danger, *B* = −0.013, SE = 0.003, CI_95%_[−0.018; −0.007]; for unscientific intervention support, *B* = 0.007, SE = 0.001, CI_95%_[0.004; 0.010]; for scientific intervention support, *B* = −0.006, SE = 0.002, CI_95%_[−0.009; −0.003]; for physical distancing, *B* = −0.011, SE = 0.005, CI_95%_[−0.021; −0.002]; and for vaccination intentions, *B* = −0.012, SE = 0.004, CI_95%_[−0.020; −0.006]. For hygiene behaviors the indirect effect was also significant but in the opposite direction, *B* = 0.013, SE = 0.006, CI_95%_[0.002; 0.025], which is due to a positive association between a belief to already have been infected and hygiene behaviors (see Table 2). For all other variables, the indirect effects and regression coefficients were consistent with our line of reasoning.

### Conspiracy mentality

We repeated the same analyses with conspiracy mentality as independent variable. The indirect effect through institutional trust was significant for perceived danger, *B* = −0.028, SE = 0.002, CI_95%_[−0.032; −0.023]; for unscientific intervention support, *B* = 0.011, SE = 0.001, CI_95%_[0.008; 0.013]; for scientific intervention support, *B* = −0.022, SE = 0.002, CI_95%_[−0.026; −0.019]; for physical distancing, *B* = −0.021, SE = 0.004, CI_95%_[−0.030; −0.013]; for hygiene behaviors, *B* = −0.028, SE = 0.005, CI_95%_[−0.038; −0.018]; and for vaccination intentions, *B* = −0.039, SE = 0.003, CI_95%_[−0.046; −0.034]. The indirect effect through self-perceived infections was significant for perceived danger, *B* = −0.003, SE = 0.001, CI_95%_[−0.004; −0.002]; for unscientific intervention support, *B* = 0.002, SE = 0.0003, CI_95%_[0.001; 0.003]; for scientific intervention support, *B* = −0.001, SE = 0.0004, CI_95%_[−0.002; −0.001]; for physical distancing, *B* = −0.003, SE = 0.001, CI_95%_[−0.005; −0.001]; and for vaccination intentions, *B* = −0.003, SE = 0.001, CI_95%_[−0.005; −0.002]. For hygiene behaviors, the indirect effect was again significant but in the opposite direction, *B* = 0.003, SE = 0.001, CI_95%_[0.001; 0.005]. The results for conspiracy mentality mirror those for the specific Covid-19 conspiracy theories.

### Actual infections

The analyses above reveal that Covid-19 conspiracy beliefs and conspiracy mentality are associated with an increased belief to already have been infected with the coronavirus. We also examined whether conspiracy beliefs were related with actual infections, as indicated by a positive medical test. Our weighted sample contained 165 participants who had received a positive medical test. These participants did not differ from the rest of the sample in conspiracy mentality, however, *t*(7188) = −0.63, *p* = 0.528; *d* = 0.06, and reported *lower* Covid-19 conspiracy beliefs (M = 1.32, SD = 0.18) than the rest of the sample (M = 1.85, SD = 0.70), *t*(7573) = 9.89, *p* < .001; *d* = 1.05. These findings do not support the possibility that—at least early in the pandemic, when we conducted this study—the link between conspiracy beliefs and self-perceived infections is due to actual infections; instead, they are consistent with the idea that conspiracy beliefs are associated with an increased tendency to interpret mild physical symptoms as evidence of a Covid-19 infection.

## Discussion

The present study sought to uncover a previously unrecognized mediator of the link between conspiracy beliefs and health beliefs and behaviors in the context of the Covid-19 pandemic: Conspiracy beliefs are related with the belief to already have been infected with the virus. This observation is consistent with the underlying theoretical idea that conspiracy beliefs are rooted in a suspicious mindset that is also relatively vigilant toward one’s own physical signals, reinforcing beliefs such as the one that Covid-19 produces only mild symptoms. Moreover, independent of the well-established role of institutional distrust (Freeman et al., 2020; Karić and Međedović, 2021; Pummerer et al., 2021; Swami et al., 2011), self-perceived infections accounted for the relationships of conspiracy beliefs with a decreased perception of the virus as dangerous, increased support for non-scientific interventions, decreased support for scientific interventions, decreased physical distancing, and decreased vaccination intentions.

A possible alternative explanation for the link between conspiracy beliefs and self-perceived infections is that conspiracy beliefs predict an increased likelihood of actually getting infected. While this idea has merit in the long run (i.e. it is plausible that due to decreased compliance with containment-related behavior, conspiracy beliefs predict a higher likelihood of eventually getting infected), it cannot explain the present pattern of results, for two reasons. First, the data were collected very early in the pandemic, and it is likely that, at least at the level of individual respondents, the link between containment-related behavior and actual infection outcomes becomes statistically apparent only in the longer run (Fazio et al., 2021). Second, we measured actual infections, and the results did not reveal that conspiracy beliefs predict a higher likelihood of a positive Covid-19 medical test. In fact, the results revealed the contrary, suggesting that actually experiencing a medically confirmed case of Covid-19 may reduce people’s belief in some of the conspiracy theories surrounding the pandemic.

The present line of reasoning was supported for all dependent variables except hygiene behaviors: Although Covid-19 conspiracy beliefs and conspiracy mentality were (weakly) negatively correlated with hygiene behaviors, this relationship was mainly accounted for by the path through institutional trust. Self-perceived infections *positively* predicted increased hygiene behaviors in the mediation models. A possible explanation is that independent of one’s conspiracy beliefs or institutional trust, a self-perceived infection with a communicable disease reminds people of the value of personal hygiene.

The study has a number of noteworthy strengths and limitations. Strengths are the large sample, that was weighted to provide representative estimates of the Dutch adult population. Hence, although the current contribution offers only a single study, it does provide a powerful test of the current line of reasoning on high-quality data. A limitation is the cross-sectional design, making it impossible to draw conclusions about cause and effect. While some studies do show causal effects of conspiracy beliefs on Covid-19 health beliefs and behaviors (Pummerer et al., 2021), there are also theoretical arguments supporting the opposite causal order: People may use conspiracy theories to justify their existing beliefs and behaviors during the pandemic (Mercier, 2020; cf. Festinger, 1957). Hence, experimental and longitudinal designs are necessary to more precisely uncover the causal processes that our model assumes.

A second limitation is that the present data only provide evidence for part of the psychological process that we assume. While the results support the idea that conspiracy beliefs predict an increased self-perceived chance of already having been infected, the present study does not provide direct evidence for the underlying theoretical line of reasoning to account for this link. Future research may therefore more directly investigate if conspiracy beliefs predict a higher likelihood of (potentially unwarranted) suspicion toward their own body, in the form of interpreting mild physical symptoms as evidence for a Covid-19 infection. Also, future research may examine additional mediators for the link between conspiracy beliefs and health-relevant beliefs and behaviors.

To conclude, the Covid-19 pandemic has inspired many conspiracy theories, and among scientists and policy-makers there is growing consensus that belief in such theories is not harmless: Conspiracy beliefs have genuine implications for public health, for instance through decreased support for lockdown policies, reduced physical distancing, and reduced vaccination intentions. While showing that these relationships exist is important, explaining *why* they emerge is quite another challenge. The present paper makes a contribution to these issues by providing evidence that besides institutional distrust, conspiracy beliefs also predict an increased belief to have already have been infected, which contributes to people’s health beliefs and behaviors. Apparently, the mindset associated with conspiracy thinking has implications beyond how suspicious people are toward other people or groups: It also predicts how people interpret the physical signals coming from their own body.

## Supplemental Material

sj-docx-1-hpq-10.1177_13591053211051816 – Supplemental material for Just a Flu? Self-perceived infection mediates the link between conspiracy beliefs and Covid-19 health beliefs and behaviors Click here for additional data file. Supplemental material, sj-docx-1-hpq-10.1177_13591053211051816 for Just a Flu? Self-perceived infection mediates the link between conspiracy beliefs and Covid-19 health beliefs and behaviors by Jan-Willem van Prooijen, Tom W Etienne, Yordan Kutiyski and André PM Krouwel in Journal of Health Psychology

## Notes

1. The questionnaire also contained a different, 4-item measure of Covid-19 conspiracy beliefs on an 11-point scale. Analysis of this scale yielded similar results, which are available upon request.

2. See link: https://osf.io/wbajq/
