# Supplementary material for: Just a Flu? Self-perceived infection mediates the link between conspiracy beliefs and Covid-19 health beliefs and behaviors
Source: J Health Psychol. 2021 Oct 20;27(6):1421–31. doi: 10.1177/13591053211051816 (PMC9036149; doi:10.1177/13591053211051816)
Supplement: sj-docx-1-hpq-10.1177_13591053211051816 – Supplemental material for Just a Flu? Self-perceived infection mediates the link between conspiracy beliefs and Covid-19 health beliefs and behaviors [file sj-docx-1-hpq-10.1177_13591053211051816.docx]

Online Supplementary Materials

for

**Just a Flu? Self-perceived Infection Mediates the Link between Conspiracy Beliefs and Covid-19 Health Beliefs and Behaviors**

**Measures**

**Note**

All stimulus materials were in Dutch, and the measures reported here were part of a larger questionnaire. Here, we provide the English translations of the measures reported in the paper; original Dutch wordings, and information about other variables assessed, are available upon request.

**Covid-19 Conspiracy beliefs**

How credible do you find the below statements about the corona virus? (1 = not very credible, 5 = very credible)

- The virus has been released by the US government to destabilize China
- The virus was developed to control population growth
- The virus is a way to cover up the effects of 5G towers
- The virus was developed by pharmaceutical companies
- Together with the future vaccine, a chip will be injected to permanently track people
- The Chinese government lies about the number of corona deaths in China
- The corona virus was spread deliberately among the Chinese population
- The corona virus was developed by the Chinese government to damage the Western world and its economies, in order to become the strongest economy in the world
- The corona virus was developed by climate activists to counteract climate change

**Conspiracy mentality (Bruder et al., 2013)**

*Responses were on 1 (certainly not 0%) to 11 (certainly 100%)*

- I think that many very important things happen in the world, which the public is never informed about
- I think that politicians usually do not tell us the true motives for their decisions
- I think that government agencies closely monitor all citizens
- I think that events which superficially seem to lack a connection are often the result of secret activities
- I think that there are secret organizations that greatly influence political decisions

**Trust in institutions:**

Can you tell how much trust you have in each of the below institutions and countries^[[1]](#footnote-1)^? (1 = Not at all, 4 = A lot)

- The army
- The educational system
- Media
- Labor unions
- The police
- Parliament
- Public employees
- The social security system
- The European Union
- The NATO
- The judicial system
- Big Dutch companies
- Multinational companies
- Environmental organizations
- Political parties
- The health care system

**Infections**

***Self-perceived infections:***

- How big do you consider the chance to already be (or have been) infected with the corona virus? (1 = *very small*; 5 = *very big*)

***Actual infections:***

- Did you test positively for the corona virus (Covid-19); meaning that you (now or earlier) have experienced a medically confirmed case of this disease? (*yes* / *no*)

**Health beliefs:**

***Perceived danger:***

*Responses on a scale (1 = certainly not, 5 = certainly)*

- It is dangerous to get infected with the corona virus
- Do you believe that many citizens will die as a consequence of Covid-19?
- Do you believe that the media exaggerate the dangers of Covid-19? (recoded)

***Unscientific intervention support:***

How effective do you believe the following measures are to fight the corona virus? (1 = not at all effective, 5 = very effective)

- Ignore the virus and continue life as usual
- Praying
- Rely on herbal medicine or other alternative treatments
- Breathe in warm vapors
- Blow a hair dryer in your nose
- Malaria medication (such as hydroxychloroquine)
- Gargle with warm water
- Take a high dose of vitamin C

***Scientific intervention support:***

How effective do you believe the following measures are to fight the corona virus? (1 = not at all effective, 5 = very effective)

- Prohibit public gatherings
- Stay indoors as much as possible
- Wear a face mask
- Wash hands regularly
- Close down public places (e.g., restaurants, schools)
- Scale up the capacity of Intensive Care Units in hospitals
- Develop a vaccine against corona
- Keeping 1.5 meters distance from each other

**Health behaviors:**

***Physical distancing:***

*Responses on a slider, ranging from 0 = strongly disagree, 10 = strongly agree*)

During the days of the corona pandemic……

- I stay at home as much as possible
- I have visited friends, family or colleagues outside my home (recoded)
- I limit the number of visits to the supermarket to an absolute minimum
- I keep physical distance from other people outside my home
- I avoid shaking hands with people outside my home

***Personal hygiene:***

*Responses on a slider, ranging from 0 = strongly disagree, 10 = strongly agree*)

During the days of the corona pandemic……

- I wash my hands longer than normal
- I wash my hands (with soap) more thoroughly than normal
- I wash my hands always directly upon arriving at home
- I disinfect often-used objects, such as mobile phones and keys
- I cough and sneeze in my elbow

***Vaccination intention:***

- Would you get vaccinated with a future vaccine against Covid-19? (1 = *certainly not*, 5 = *certainly*).

1. The items also included “The US” and “Russia”; however, we dropped these two items as they loaded on a different factor in a factor analysis. [↑](#footnote-ref-1)
